# Direct Growth and Controlled Coalescence of Thick AlN Template on Micro-circle Patterned Si Substrate

**DOI:** 10.1038/srep14734

**Published:** 2015-10-06

**Authors:** Binh Tinh Tran, Hideki Hirayama, Noritoshi Maeda, Masafumi Jo, Shiro Toyoda, Norihiko Kamata

**Affiliations:** 1 Quantum Optodevice Laboratory, Institute of Physical and Chemical Research (RIKEN), 2-1 Hirosawa, Wako, Saitama 351-0198, Japan

## Abstract

High-density micro-circle patterned Si substrates were successfully fabricated for the direct overgrowth of thick AlN templates by using NH_3_ pulsed-flow multilayer AlN growth and epitaxial lateral overgrowth techniques. The experimental results show that an 8-μm-thick AlN template was grown at a very high growth rate on the substrates. The AlN template had full widths at half maximum of 0.23° and 0.37° for the (002) and (102) reflection planes in X-ray diffraction rocking curves. Atomic force microscopy and transmission electron microscopy confirmed that the roughness of the surface was low (3.5 nm) and the dislocation density was very low (1.5 × 10^8^ cm^−2^ (screw), 3.7 × 10^8^ (edge) cm^−2^).

AlGaN-based deep ultraviolet light-emitting diodes (UV-LEDs) have attracted considerable attention due to their wide range of applications in air and water purification, disinfection, chemical sensing, biomedicine, and non-line-of-sight communication^1^,^2^. AlGaN-based deep UV-LEDs on sapphire substrates are now commercially available. However, AlGaN growth on silicon substrates is challenging to achieve, and has been extensively studied. AlGaN-based deep UV-LEDs require a thick, high-quality AlN template to be grown on the Si substrate before further AlGaN layers can be grown. Because the AlN template can transmit very short wavelengths (~210 nm), the Si substrate can be removed by chemical treatment to allow back illumination, and avoid the generation and reabsorption of UV light by backside emission^3^,^4^,^5^. Good-quality AlN templates are crucial for obtaining high-efficiency AlGaN-based deep UV-LEDs. However, there are many difficulties in growing a thick AlN template on Si substrates: the large lattice mismatch between AlN and Si(111) (~23.4%)^6^ causes high dislocation density and crack initiating stress; the presence of the native oxide layer on the Si substrate leads to low coherence between the AlN template and the Si substrate^7^,^8^; AlN species with low mobility on the Si surface inhibit the structural rearrangement^9^; and the low growth rate, which is the main problem preventing the development of AlN films on Si and sapphire substrates. Thus, the conventional growth of bulk AlN on Si substrates is a major problem for researchers.

AlN nucleation layers deposited on Si substrate by metal organic chemical vapor deposition (MOCVD) reactor at low temperatures typically show a very low growth rate and a mosaic structure with very high threading dislocation of about 10^9^–10^11^ cm^−2^, for a very thin AlN template (≤1 μm)^10^,^11^. Therefore, various methods have been used to suppress these problems for AlN templates growth, such as native bulk AlN substrates, migration enhanced MOCVD growth, pulsed-flow multilayer AlN growth, growth mode modification, and high-temperature growth^12^,^13^,^14^,^15^,^16^ on stripe patterned AlN/Si or AlN/sapphire substrates^5^,^17^,^18^,^19^. There are to our knowledge no reports of the direct growth of thick AlN templates on micro-circle patterned Si substrates (mPSiS).

In this work, we report the fabrication of mPSiS and investigate thick AlN templates grown directly on these substrates for the first time. We used NH_3_ pulsed-flow multilayer AlN growth and epitaxial lateral overgrowth (ELO) to obtain templates suitable for AlGaN-based deep UV-LEDs in the future. The effect of the growth temperature of the initial AlN layer on the crystal quality and the relationships among the V/III ratio, AlN growth rate, and crystalline quality of the thick AlN templates were determined. The samples were characterized by using X-ray diffraction (XRD), atomic force microscopy (AFM), scanning electron microscopy (SEM), and transmission electron microscopy (TEM) to determine the crystalline quality, surface roughness, thickness, and dislocation density, respectively.

## Results and Discussion

**Figure 1:**
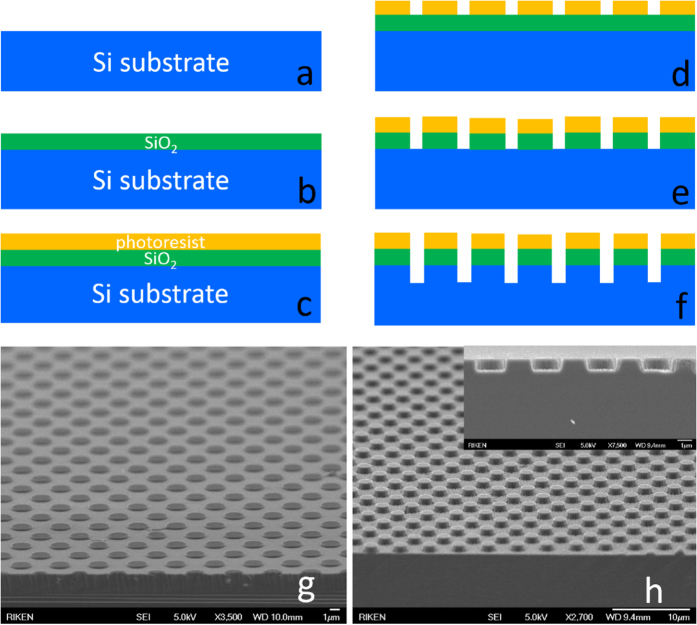
Steps for fabricating a mPSiS by using standard photolithography and ICP etching techniques (a–f). Si substrate after treated with a BOE (**a**), deposited SiO_2_ by PECVD (**b**) and coated a photoresist layer (**c**). Using standard photolithography to lithograph the mask (**d**), then etched the mask (SiO_2_) (**e,g**) and Si substrate (**f **). Finally, Si substrate etched (mPSiS) with SiO_2_ has been removed (inset shows cross-sectional image) (**h**).

The SiO_2_ (250 nm thick) mask layer was deposited on the 2 in. Si(111) substrates by using plasma-enhanced chemical vapor deposition system (SAMCO CVD PD-220 N). Substrates were subjected to standard lithography, and inductively coupled plasma (SAMCO ICP RIE-200iP) was used to etch the SiO_2_/Si substrates layer by layer. For SiO_2_ etching, CF_4_ was supplied at 20 sccm for 2.5 min. Si was etched with CF_4_:O_2_ (10:1) for 50 min at 100 Torr and a 10 W bias. The SiO_2_ mask used for patterning and the photoresist were completely removed by using acetone and wet chemical etching (buffered hydrofluoric acid) before AlN overgrowth. The fabrication of the substrate and the final mPSiS are shown in Fig. 1(a–h). The micro-circle pattern had diameters of about 1.5 μm, a depth of 1 μm, and a period of 3.5 μm.

**Figure 2:**
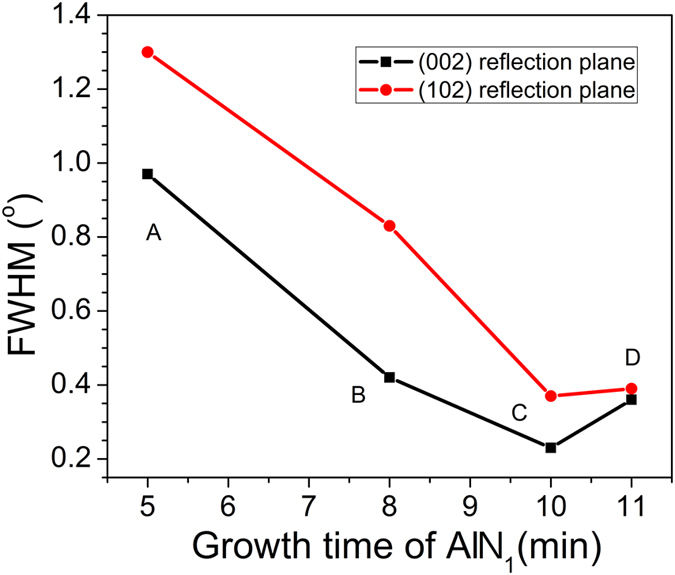
FWHMs of the XRD rocking curves in the symmetric (002) and asymmetric (102) planes of AlN templates grown with different first AlN layer growth times.

Figure 2 shows summarizes the full width at half-maximum (FWHM) of the XRD rocking curves of the AlN templates with the first AlN layer grown for different times. The FWHMs of the (002) and (102) reflection planes of the AlN templates decreased with increasing growth time of the first AlN layer from 5 to 10 min and slightly increased with the increase of the growth time from 10 to 11 min. The lowest FWHM value was obtained at a growth time of 10 min in sample C with values of 0.23° (002) and 0.37° (102), compared with 0.97° (002) and 1.30° (102) for sample A, 0.42° (002) and 0.83° (102) for sample B, and 0.36° (002) and 0.39° (102) for sample D. Thus, a suitable growth time for the first AlN layer enabled a reduction in AlN/mPSiS threading dislocations by up to about 4 times. A high growth rate was important for obtaining these results^11^. Sample C was grown at a rate of more than 50 nm/min with a V/III ratio of about 150, which was a very high growth rate compared with recent studies^11^,^19^,^20^,^21^. The details of V/III ratio and growth rate will be discussed later.

**Figure 3:**
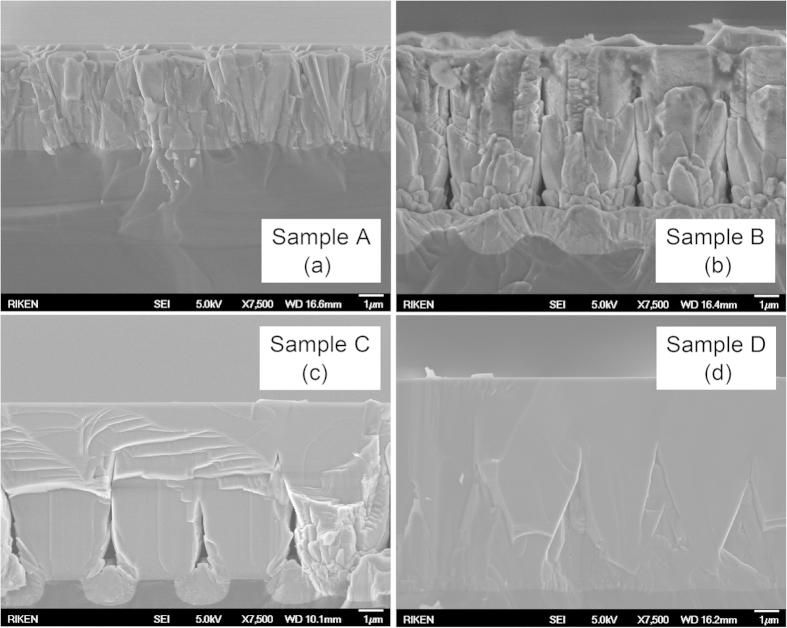
Cross-sectional SEM images show the excellent coalescence thickness of sample C compared with samples A, B, and D.

Figure 3 shows cross-sectional SEM images of samples A–D. Samples A and B, which had a first AlN layer grown for 5 and 8 min, respectively, revealed that the both surfaces were either facetted and rough, or no coalescence thickness was achieved (Fig. 3(a,b)). For samples C and D, coalescence thickness was achieved and increased quickly for the two samples with smooth surfaces as confirmed by AFM measurements. However, the XRD results confirmed that the crystallinity of sample C was higher than that of sample D, which may be caused by the surface of sample D being worse than that of sample C. The surface roughness of samples C and D were 3.5 and 7.7 nm, respectively, as measured by AFM (images not shown), whereas samples A and B had surface roughnesses of 132 and 98 nm, respectively. In addition, there were no cracks on the surface of all samples were observed, even though they all were grown at very high temperatures, which usually causes cracking during cooling because of thermally induced tensile stress^7^.

**Figure 4:**
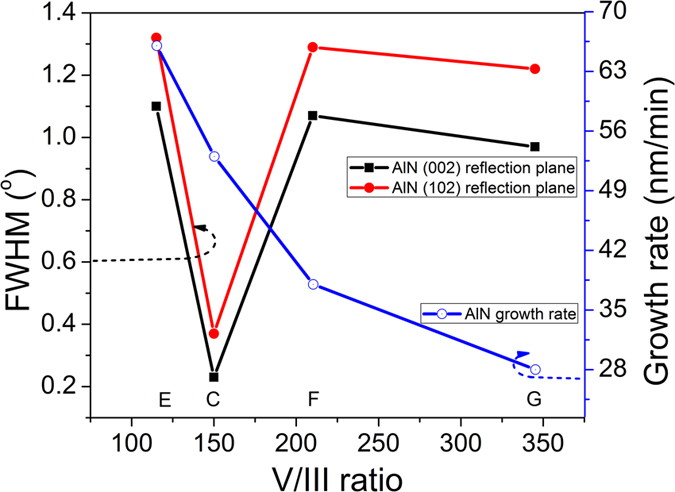
Vertical growth rate as a function of the V/III ratio (blue line). At a V/III ratio of 150 in sample C, a very low FWHM has been obtained.

For investigation of the V/III ratio versus the growth rate and the crystalline quality (FWHM), the next four samples had different V/III ratios of 115, 150, 210, and 345 and named samples E, C, F, and G, respectively. Samples E, F, and G were grown under the same conditions as sample C, except for the V/III ratio, which was controlled by the TMAl flow. Figure 4 shows the V/III ratio versus the FWHM and the growth rate of the AlN template. The vertical growth rate of the AlN template shown as a function of V/III ratio increases with the decrease in the V/III ratio (blue line). The highest growth rate was 66 nm/min, which is very high compared with other recently reported growth rates^11^,^19^,^20^,^21^. However, the FWHM was not a function of the V/III ratio. Around a V/III ratio of 150 in sample C, the FWHM increased quickly. The FWHM profile was similar to that of the AlN template grown on stripe patterned AlN/sapphire^19^, although the variance of the FWHM profile was larger. This may be caused by the direct growth of the AlN template on the Si substrate.

**Figure 5:**
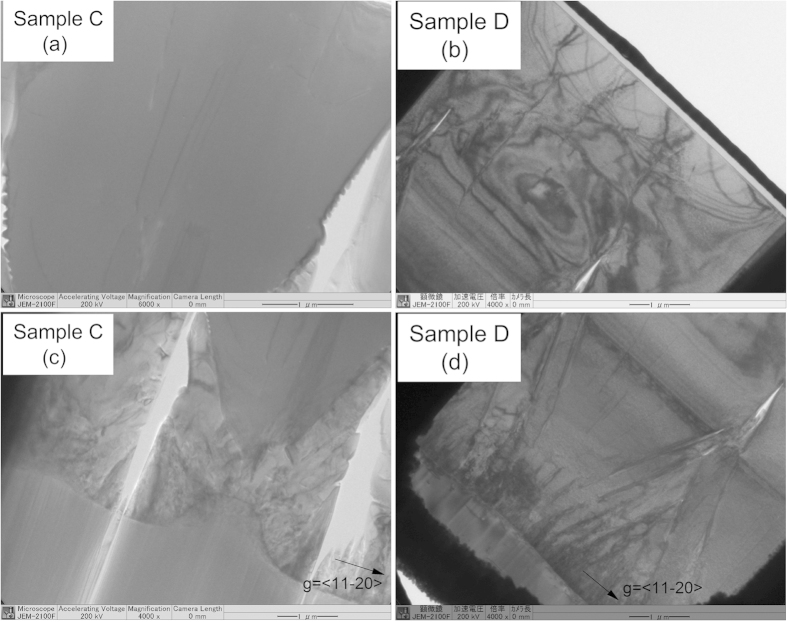
Cross-sectional TEM images of the ELO AlN templates with a growth time for the first AlN layer of 10 and 11 min on mPSiS of samples C and D, respectively. (**a–d**) show the threading dislocation at top and bottom of each sample.

To understand the effect of the dislocation density of the thick AlN template grown on mPSiS, we performed TEM measurement using the JEOL JEM-2100F. The bright-field cross-sectional TEM images were obtained with g = [11–20] for samples C and D as shown in Fig. 5. The TEM images only show a few threading dislocations in a large scanned area in the middle of the cross-sectional AlN template of sample C (Fig. 5(a,c)). The dislocations were mostly distributed at the bottom of the template and were bent towards the sidewall of voids and terminated at the sidewall (Fig. 5c), owing to the ELO technique. While, the coalescence thickness of sample D was not built well with no voids as we can observe from the Figs 3(d) and 5(d). The dislocation density of sample D is also higher than that of sample C as can be seen in Fig. 5. Thus, a not suitable growth time of the first AlN which may be caused and led to poor subsequent ELO layers. However, these TEM images confirmed that there were no gaps in the coalescence thickness in both samples C and D. The dislocation densities estimated using TEM images around the middle region of the AlN templates were approximately 1.5 × 10^8^ cm^−2^ (screw) and 3.7 × 10^8^ cm^−2^ (edge) for sample C. These values were low as compared with about 4.2 × 10^8^ cm^−2^ (screw) and 9.2 × 10^8^ cm^−2^ (edge) for sample D with only 1 min difference in growth times of the first AlN layer. These dislocation densities indicated that the growth time of the first AlN layer is significantly important for successfully obtaining high-crystallinity thick AlN templates grown on mPSiS.

## Conclusions

We fabricated high-density mPSiS and investigated 8-μm-thick fully coalesced AlN templates directly overgrown on these substrates by NH_3_ pulsed-flow multilayer AlN growth and ELO techniques. The effect of the thickness of the initial AlN layer and the V/III ratio were also investigated. The thickness of the initial AlN layer was very important, and the coalescence thickness was achieved quickly when the growth time for the initial AlN layer was more than 8 min. Selection of appropriate initial AlN growth parameters controlled the coalescence thickness of the thick AlN template. The growth rate could be adjusted by controlling the V/III ratio. At a very high growth rate (50 nm/min) and a V/III ratio of about 150, the highest crystallinity of AlN template with the dislocation density in the order of 10^8^ cm^−2^ was obtained. The direct growth of the AlN template on high-density mPSiS achieved a good crystallinity compared with stripe patterned AlN/Si substrate as mentioned above. Moreover, our technique is faster and cheaper because it requires fewer steps; it is not necessary to pre-grow and pattern a thin AlN layer on Si substrate before overgrowing the AlN template. Thus, this AlN template technique is promising for growth of AlGaN-based deep UV-LEDs.

## Methods

AlN templates were grown on Si(111) substrates by using a low-pressure MOCVD rotating disk vertical reactor. Trimethylaluminum (TMAl) and NH_3_ were used as sources for Al and N, respectively. Before pattern fabrication, the 2 in. Si(111) substrates were treated with a buffered oxide etchant, and then rinsed with deionized water for 5 min to remove the surface oxide layer. The Si substrates were then sent for pattern fabrication as detailed above. The total thickness of each AlN template included five AlN layers that were grown by using the NH_3_ pulsed-flow multilayer AlN growth and ELO techniques. For samples A, NH_3_ pulsed-flow multilayer AlN growth was used to grow the first AlN (AlN_1_) layer for 5 min, and the second and fourth AlN layers (AlN_2_ and AlN_4_) for 11 and 8 min, respectively. ELO was used to grow the third and fifth AlN layers (AlN_3_ and AlN_5_) for 1 hour each. Samples A–D had a first AlN layer grown for 5, 8, 10, and 11 min, respectively. The details of growth parameters for samples A–D are shown in the Table 1.

## Additional Information

**How to cite this article**: Tran, B. T. *et al.* Direct Growth and Controlled Coalescence of Thick AlN Template on Micro-circle Patterned Si Substrate. *Sci. Rep.* **5**, 14734; doi: 10.1038/srep14734 (2015).

## Figures and Tables

**Table 1:** Samples A–D were grown with the same parameters, except for the growth time of the first AlN layer (AlN_1_).

Samples	TMAl_1–5_ flow(sccm)	Growth time(min) AlN_1–5_	Pressure(Torr)AlN_1, 2–5_	GrowthTemperature(°C) AlN_1–5_	NH_3_ (sccm)AlN_1–5_
A	10-10-35-10-35	5-11-60-8-60	200, 76	1390	10-10-5-10-5
B	10-10-35-10-35	8-11-60-8-60	200, 76	1390	10-10-5-10-5
C	10-10-35-10-35	10-11-60-8-60	200, 76	1390	10-10-5-10-5
D	10-10-35-10-35	11-11-60-8-60	200, 76	1390	10-10-5-10-5

The growth times of the other layers were optimized. For samples E, F, and G, they were grown under the same conditions as sample C, except for the V/III ratio by controlling the TMAl flow and not listed here.
